# Remarks on Treatment of Fractures

**Published:** 1850-05

**Authors:** 


*Remarks on the Treatment of Fractures.* By L. A. DUGAS, M. D., Prof. of Physiology, &c., in the Medical College of Georgia.

Although it may seem a work of supererogation to write upon a subject so trite and so familiar to every practitioner as that of Fractures, there are still some points upon which surgeons differ, and upon which they should therefore be heard. On the general principles of treatment all agree; but such is not the case when we come to the details. For instance, the application of a roller bandage to the fractured limb is recommended by many and condemned by others. Among the former, there some who advise its immediate application, and others who defer it until the inflammatory stage shall have passed. In our country the bandage is, I believe, very generally used, and it is for this reason I propose to offer a few remarks against the propriety of such a course.

What are the ends proposed to be attained by the application of the roller or other compressing bandage to a fractured limb? They are, I believe, three-fold, viz: to aid in retaining the bones in their proper adaptation, to prevent the swelling of the limb, and to reduce this after it has occurred. A serious objection to the bandage thus used, is that its *appliplication* constitutes by far the most painful portion of the dressing, especially if the limb be held for the purpose by an unskilful aid, No one who has ever witnessed the application of the roller bandage, from the toes up to the pelvis, in fractures of the os femoris, when every turn of the roller, however gently carried, imparts motion and intense pain, can have failed to wish that it might be dispensed with. Such, at least, is the case with the patient, if not with the surgeon. This evil is aggravated by the necessity, which very soon occurs, of removing and re-applying the bandage, as will be hereafter stated. Now, if the proper apposition of the fractured ends can be secured *without* the roller bandage, is not the difficulty and painfulness of its application a sufficient reason to abandon it? But it is also proposed to prevent, by its use, the development of swelling. Let us see if this object is ever attained. Every one knows how difficult it is to apply the roller bandage to a whole limb in such a way that the compression will be perfectly uniform, and the circulation not impeded.

Even expert surgeons sometimes fail in this, and the less experienced will, of course, do so still more frequently. But, however skilfully applied, the tendency to swelling *at the seat of fracture,* will very soon make the bandage more tight at this point than below it: the venous circulation will become impeded; pain will supervene and increase until the patient or his friends will be compelled to cut loose the bandage, in order to release the stricture. The patient will then have to remain with an imperfect adjustment of dressings until the physician can see him, which, in the country, may be, not only hours, but days. Cases also unfortunately occur occasionally, in which, from the docility or fortitude of the patient, he does not demand and obtain timely relief from the compression, and suffers mortification to take place. One of the most distinguished surgeons of the North stated to the writer, a few months since, that he had been re peatedly called upon to perform amputation, in consequence of the tightness of the bandage occasioned by the supervention of swelling at the seat of fracture. There can be no doubt that such accidents are much more common than generally supposed, from the fact that few men are as fond of reporting their unfortunate cases, as they are of heralding their successful achievements.

The third object proposed to be attained by the roller bandage, is the reduction of the swelling or tumefaction usually occasioned by fractures. For this purpose, the bandage is advised to be applied *after* the tumefaction shall have reached its maximum. At this stage of the case, the bandage is unquestionably less objectionable than it is at an earlier period; yet its application, even now, is very painful; it is still difficult; and it may be so applied as to produce unequal pressure and consequent strangulation, with all its inconveniences and dangers. If it were absolutely necessary, these objections might be waived; but, if not, they should have their full weight in determining the practice to be adopted. It is certainly not *absolutely* necessary thus to reduce the swelling; and the utility of the reduction by such means is extramely questionable. That any real evil arises from such tumefaction as usually follows fractures, has yet to be demonstrated. If left to the efforts of nature, it will subside in due time, without the use of any compression whatever.

If the bones can be maintained in apposition, and the swelling be subdued without the roller bandage, and if this bandage cannot, without great danger, be depended upon for the *prevention* of tumefaction, the necessary inference is that it may be omitted without impropriety. If, again, it be true that the manipulations required for the application of the roller bandage are always painful, that they have almost invariably to be repeated once or more as the swelling progresses, that the compression is generally the principal cause of pain in the treatment of fractures, and that it occasionally induces mortification when least expected, we should conclude, not only that it may be ommitted without impropriety, but that its use ought to be abandoned in general practice.

The writer wishes not to be understood as alluding here to the starch bandage recommended by the distinguished surgeon of Brussels. The number of victims to its use when first suggested, remains yet to be told. Suetin, however, no longer calls it the "immovable bandage," but the "*movable and immovable bandage,*" and, so great is his apprehension that the roller bandage, which constitutes a part of it, may be applied with a view to compression, and therefore perhaps too tightly, that he advises a bit of tape to be placed longitudinally along the two sides of the limb before the roller bandage, and in such a manner that the ends will project above and below: the roller bandage is then to be applied with only as much tightness as may be required to keep it in place; after which, the ends of the tape are to be drawn upon, for the purpose of ascertaining by their freedom of motion, that the compression is neither too great nor unequal. If much swelling ensue, it will be manifested, not only by pain and the appearance of the distal end of the limb, which is always to be left exposed for inspection, but also by the difficulty of moving the tapes beneath the bandage; in which events he urges the bandage to be slit open and readjusted more loosely. With these abundant precautions, upon which Suetin now dwells with great earnestness, the plan is unquestionably the best that can be devised, whenever the patient can have ready access to the surgeon or to an expert nurse as soon as it may become necessary to modify the dressing.

In establishing rules of practice, whether in medicine or in surgery, authors do not sufficiently discriminate between the various circumstances in which both practitioners and patients may be situated. What may be easy and proper under certain circumstances, may prove difficult and injudicious under a different state of things. A system of practice may be highly beneficial and unobjectionable in hospitals or cities, and be entirely unsuited to the camp or country. What may be harmless in the hands of highly cultivated and experienced physicians, may cease to be so under the administration of practitioners less skillful. It is therefore important that the principles as well as the details of general practice, be plain, intelligible to all, and of easy execution. The safety of society demands that dangerous expedients be discountenanced by the profession, especially whenever more harmless procedures can be substituted for them. The indiscriminate use of the roller bandage in the treatment of fractures, has often occasioned the most serious accidents, and should give way to the simple use of splints and bandages applied in such a manner as to admit of being modified, according to the progress of tumefaction, by any person of ordinary intelligence. Let the more complicated and hazardous processes be confined to such cases as may be continually under the supervision of the surgeon.—*Southern Medical and Surgical Journal.*

